# The Use of Romhilt-Estes Criteria in the Presumptive Electrocardiographic Diagnosis of Left Ventricular Hypertrophy in Comparison to Voltage-Based Criteria

**DOI:** 10.7759/cureus.28003

**Published:** 2022-08-14

**Authors:** Mohamed Hamed, Gopika Dasari, Joel A Casale, Navneet Kaur, Mitchell Karl

**Affiliations:** 1 Internal Medicine, Florida Atlantic University Charles E. Schmidt College of Medicine, Boca Raton, USA; 2 Pulmonology and Critical Care, University of Southern California, Los Angeles, USA; 3 Cardiology, Florida Atlantic University Charles E. Schmidt College of Medicine, Boca Raton, USA

**Keywords:** ecg (electrocardiogram), ekg abnormalities, romhilt-estes score system, concentric lvh, left ventricular hypertrophy (lvh), lvh

## Abstract

Background

The ECG diagnosis of left ventricular hypertrophy (LVH) has been challenging for over a hundred years. ECG diagnosis of LVH has shown good specificity but lacks sensitivity. In addition, voltage-based criteria can be affected by multiple conditions such as obesity and chronic lung disease. Therefore, we sought to compare Romhilt-Estes (R-E) criteria with commonly used voltage-based criteria in presumptive ECG diagnosis of LVH.

Methods

This is a retrospective electronic medical record study from September 1, 2017, to September 1, 2018, of 499 consecutive ECGs from Boca Raton Regional Hospital. Different ECG criteria were used to identify the presence of LVH, including the Cornell criteria, modified Cornell criteria, Sokolow-Lyon criteria, and Romhilt-Estes criteria. The main study outcome was to compare the R-E criteria in presumptive ECG diagnosis of LVH to the voltage-based criteria (Cornell, modified Cornell, and Sokolow-Lyon).

Results

After analyzing the ECGs using the different ECG criteria, R-E criteria were positive with LVH present (score ≥ 5 points) in 162 patients. In contrast, Cornell criteria were positive in 42 patients (8.4%), modified Cornell criteria in 50 patients (10%), and Sokolow-Lyon criteria in 13 patients (2.6%). In addition, R-E criteria showed higher positivity of LVH diagnosis compared to the sum of three voltage-based criteria (32.7% versus 21% respectively, p<0.001).

Conclusion

We presume that R-E criteria can help better diagnose LVH by ECG compared to other commonly-used voltage-based criteria. However, further studies are needed using confirmatory imaging to confirm the accuracy of R-E criteria and compare it with other voltage based-criteria.

## Introduction

Hypertension (HTN) is a major modifiable risk factor for cardiovascular disease. The left ventricle (LV) is a primary target for HTN end-organ damage [1]. Left ventricular hypertrophy (LVH) is growth in left ventricular mass caused by increased cardiomyocyte size, which can be physiological or pathological [2-5].

The ECG diagnosis of LVH has been a challenge for more than 100 years since it was first observed by Lewis in 1914 [6]. The original reference methods used in developing specific electrocardiographic criteria for the presence of LVH were either autopsy measurements or clinical assessments [7]. Although ECG diagnosis of LVH has shown good specificity (up to 98.8%), it did lack sensitivity (40%-60%) [8-10]. Romhilt-Estes (R-E) was first introduced in 1968 as an early effort to improve ECG's ability to detect and diagnose LVH before imaging methods [11]. R-E is mainly based on a point system that can help in the prediction of LVH, which is determined by six ECG features, and the presence of each feature has its own assigned points [12]. If the given ECG has a total score of 3 or less, it is considered unlikely to have LVH, 4 points are considered probable LVH, and a score of 5 or more is considered positive for LVH. The R-E score has proven to be more specific in predicting LVH [12]. Voltage-based LVH criteria can be affected by multiple factors, including age, sex, obesity, and chronic lung disease, limiting the prediction of LVH [10,13,14]. Therefore, we sought to compare the R-E criteria to different voltage criteria (Sokolow-Lyon, Cornell, and modified Cornell criteria) in the presumptive diagnosis of LVH.

## Materials and methods

Data source

This was a retrospective study from September 1, 2017, to September 1, 2018. A total of 499 consecutive ECGs from Boca Raton Regional Hospital, a 400-bed tertiary hospital, were identified and analyzed. Therefore, this study was exempted by Institutional Review Board (IRB), as it was a retrospective study from medical records with de-identified data.

Selection criteria

Inclusion criteria are any patient with an ECG for any reason. Exclusion criteria are any patient under the age of 30 as LVH detection with voltage criteria has not been well established in this age group. ECGs with complete left bundle branch block or ventricular paced rhythm were also excluded from the study.

Data extraction and analysis

ECGs were read by three investigators (Gopika Dasari, Joel A. Casale, Navneet Kaur) independently who analyzed all the ECGs for LVH by either one of the voltage criteria (Cornell, modified Cornell, or Sokolow-Lyon), R-E criteria, or both. Charts were identified for any comorbid illnesses, including asthma, chronic obstructive pulmonary disease (COPD), coronary artery disease (CAD), congestive heart failure (CHF), and renal insufficiency/failure. Statistical significance was considered for a p-value<0.05.

Identification of LVH by different criteria is outlined in Table 1, and the R-E score is identified in Table 2.

**Table 1:** Different LVH criteria. mm: millimeter; R-E: Romhilt-Estes; LVH: Left ventricular hypertrophy.

Different LVH criteria	
Sokolow-Lyon criteria	SV1 + RV5 or RV6 >35 mm
Cornell criteria	SV3 + RaVL >28 mm for men and >20 mm for women
Modified Cornell criteria	R wave in aVL ≥11 mm
Romhilt-Estes criteria	unlikely if R-E score <4 points, likely if =4 points, and present if ≥ 5 points

**Table 2:** Romhilt-Estes score. mV: millivolt; s: seconds; ms: millisecond.

Romhilt–Estes score	
Amplitude: R or S wave in limb leads ≥ 2.0 mV or S wave in V1 or V2 ≥ 3.0 mV or R wave in V5 or V6 3.0 mV	3 points
ST-T segment pattern: ST-segment depression in opposite direction to QRS complex	
Without digitalis	3 points
With digitalis	1 point
Left atrial involvement: Terminal negativity of the P wave in lead V1 ≥ 0.01 mV and ≥ 0.04 s.	3 points
Left axis deviation QRS axis ≥ − 30°	2 points
QRS duration ≥ 90 ms.	1 point
Intrinsicoid deflection (Q-R interval) ≥ 50 ms in V5 or V6	1 point

Outcomes

The main study outcome was the ability of R-E criteria to better predict LVH compared to Cornell criteria, modified Cornell criteria, or Sokolow-Lyon criteria.

## Results

Baseline characteristics

The baseline characteristics were identified in Table 3. The mean age was 71.1 (±16.4), with a mean BMI of 26.7 ± 5.7.

**Table 3:** Baseline characteristics. COPD: Chronic obstructive pulmonary disease; CAD: Coronary artery disease; HTN: Hypertension; DM: Diabetes mellitus; CHF: Congestive heart failure.

Baseline Characteristics		
Total	499	
Age (mean) ±SD	71.1 ± 16.4	
BMI (mean) ±SD	26.7 ± 5.7	
Female	282	56.5%
Asthma	21	4.2%
COPD	45	9.0%
CAD	124	24.8%
CHF	51	10.2%
HTN	301	60.3%
DM	96	19.2%
Renal insufficiency	62	12.4%
Stroke	37	7.4%
On digoxin	11	2.2%

Outcomes

After analyzing ECGs using different ECG criteria, R-E criteria were positive, with LVH present (score ≥ 5 points) in 162 patients (32.7%) and likely (score=4 points) in 51 patients (10.3%) (Table 4). Cornell criteria were positive in 42 patients (8.4%), modified Cornell criteria in 50 patients (10%), and Sokolow-Lyon criteria in 13 patients (2.6%) (Table 5). We used LVH present of R-E criteria (score ≥ 5 points) to compare it with voltage-based criteria. R-E criteria showed a higher presumptive diagnosis of LVH than total voltage-based criteria (32.7% versus 21%, respectively, p<0.001).

**Table 4:** Romhilt-Estes criteria results.

	Unlikely (<4 points)	Likely (4 points)	Present (≥ 5 points)
Number (n)	282	51	162
%	57%	10.30%	32.70%

**Table 5:** Different LVH criteria results. LVH: Left ventricular hypertrophy.

	Cornell criteria	Modified Cornell criteria	Sokolow-Lyon criteria	Romhilt-Estes criteria
Number (n)	42	50	13	162
Percentage (%)	8.40%	10%	2.60%	32.70%

## Discussion

In this retrospective study, 499 ECGs were analyzed. We compared the most commonly used ECG - LVH criteria, including Cornell, modified Cornell, and Sokolow-Lyon criteria, with the R-E criteria to see if there was an improved detection of LVH by R-E criteria. Our results suggest that R-E criteria could be a more reliable and sensitive method to diagnose LVH compared to traditional voltage-based criteria.

Although the introduction of non-invasive imaging methods (echocardiography, cardiac magnetic resonance) has shown better sensitivity and specificity [15], ECG still remains widely used as it is an inexpensive, convenient, and readily available way of determining the presence of LVH [15]. The most traditionally used ECG criterion in predicting LVH has been the QRS-based voltage criteria which commonly involves Sokolow-Lyon, Cornell, and modified Cornell criteria. However, detection of LVH by ECG can be affected by several factors, including obesity and chronic lung disease, limiting the sensitivity of voltage-based criteria in predicting LVH [10,13,14]. On the other hand, R-E criteria avoid these limitations since it utilizes multiple ECG components with a combination of non-amplitude and non-QRS elements [15].

To our knowledge, our study is the first study to compare the R-E criteria to other commonly used voltage-based criteria. R-E criteria show other benefits beyond diagnosing LVH. In 2015, Estes EH et al. found that rising levels of R-E score were associated with increased all-cause mortality. Only four components of the R-E criteria are predictive of increased mortality (P-terminal force, QRS amplitude, LV strain, and intrinsicoid deflection) [12]. Bacharova L et al. and Estes EH et al. emphasized the role of ECG as a strong predictor of future cardiovascular disease and mortality [15-16]. Another study illustrated that an R-E score ≥ 4 is associated with an increased risk of cardiovascular disease, coronary heart disease, heart failure, and stroke [16]. It also demonstrated that each of the six individual components of the R-E criteria has a unique and independent ability to predict different cardiovascular disease outcomes [16]. According to a study by Darouian N et al., R-E criteria have also shown a benefit in expecting sudden cardiac arrest if the score was ≥5, independent of echocardiographic LVH and reduced LV ejection fraction [17].

Our study is limited as it is a retrospective study in which we could only identify the available data. Additionally, we could not confirm our findings with further imaging using echocardiography or cardiac magnetic resonance. Therefore, further larger studies are needed to evaluate different ECG LVH criteria better.

## Conclusions

This study suggests that using R-E criteria could improve the ability to diagnose LVH by ECG compared to using the more common voltage-based criteria. R-E criteria have also been shown to have other benefits such as the prediction of mortality, prediction of future cardiovascular disease, including CAD, heart failure, and stroke, and expecting sudden cardiac death. Additional studies, including a larger series of ECG reviews and confirmation of LVH by R-E criteria using imaging modalities such as echocardiography, are necessary to further test the utility of R-E criteria in diagnosing LVH by ECG.
